# Enhancing Antitumor Efficacy of Heavily Vascularized Tumors by RAMBO Virus through Decreased Tumor Endothelial Cell Activation

**DOI:** 10.3390/cancers12041040

**Published:** 2020-04-23

**Authors:** Mitra Nair, Maninder Khosla, Yoshihiro Otani, Margaret Yeh, Flora Park, Toshihiko Shimizu, Jin Muk Kang, Chelsea Bolyard, Jun-Ge Yu, Yeshavanth Kumar Banasavadi-Siddegowda, Gonzalo Lopez, Balveen Kaur, Raphael E. Pollock, Tae Jin Lee, Matthew Old, Ji Young Yoo

**Affiliations:** 1 Department of Neurosurgery, University of Texas Health Science Center at Houston, Houston, TX 77030, USA; Mitra.Nair@uth.tmc.edu (M.N.); Yoshihiro.Otani@uth.tmc.edu (Y.O.); Margaret.Yeh@uth.tmc.edu (M.Y.); fsp1@rice.edu (F.P.); Toshihiko.Shimizu@uth.tmc.edu (T.S.); Jin.Muk.Kang@uth.tmc.edu (J.M.K.); Balveen.Kaur@uth.tmc.edu (B.K.); Tae.Jin.Lee@uth.tmc.edu (T.J.L.); 2 Department of Otolaryngology-Head and Neck Surgery, James Comprehensive Cancer Center, The Ohio State University Wexner Medical Center, Columbus, OH 43210, USA; mkhosla95@gmail.com (M.K.); jgyu9999@yahoo.com (J.-G.Y.); 3 Comprehensive Cancer Center–Arthur G. James Cancer Hospital and Richard J. Solove Research Institute, The Ohio State University, Columbus, OH 43202, USA; chelsea.blessing@osumc.edu; 4 Surgical Neurology Branch, National Institute of Neurological Disorders and Stroke, National Institutes of Health, Bethesda, MD 20892, USA; kumarvet@gmail.com; 5 Department of Surgery, Division of Surgical Oncology, James Comprehensive Cancer Center, The Ohio State University Wexner Medical Center, Columbus, OH 43210, USA; Gonzalo.Lopez@osumc.edu (G.L.); Raphael.Pollock@osumc.edu (R.E.P.)

**Keywords:** oncolytic herpes simplex virus-1 (oHSV), soft tissue sarcoma (STS), rapid antiangiogenesis mediated by oncolytic virus (RAMBO), angiogenesis, vasculostatin (Vstat120), tumor microenvironment (TME)

## Abstract

Vascularization is a common pathology for many solid tumors, and therefore anti-angiogenic strategies are being investigated as a therapeutic target for treatment. Numerous studies are also being conducted regarding the effects of oncolytic viruses, including Imlygic^TM^, an FDA approved oncolytic herpes simplex virus-1 (oHSV) for the treatment of highly vascularized tumors such as Kaposi sarcoma (NCT04065152), and brain tumors. To our knowledge, the effects of combining oncolytic HSV with angiogenesis inhibition on endothelial cell activation has not been previously described. Here, we tested the effects of Rapid Antiangiogenesis Mediated By Oncolytic Virus (RAMBO), an oHSV which expresses a potent anti-angiogenic gene *Vasculostatin* on endothelial cell activation in heavily vascularized solid tumors. oHSV treatment induces endothelial cell activation, which inhibits virus propagation and oncolysis in adjacent tumor cells in vitro. Consistently, this was also observed in intravital imaging of intracranial tumor-bearing mice in vivo where infected tumor endothelial cells could efficiently clear the virus without cell lysis. Quantitative real-time PCR (Q-PCR), leukocyte adhesion assay, and fluorescent microscopy imaging data, however, revealed that RAMBO virus significantly decreased expression of endothelial cell activation markers and leukocyte adhesion, which in turn increased virus replication and cytotoxicity in endothelial cells. In vivo RAMBO treatment of subcutaneously implanted sarcoma tumors significantly reduced tumor growth in mice bearing sarcoma compared to rHSVQ. In addition, histological analysis of RAMBO-treated tumor tissues revealed large areas of necrosis and a statistically significant reduction in microvessel density (MVD). This study provides strong preclinical evidence of the therapeutic benefit for the use of RAMBO virus as a treatment option for highly vascularized tumors.

## 1. Introduction

Angiogenesis—the formation of new blood vessels—is a well-established property of solid tumors and is essential for their growth and metastasis [1]. Multiple efforts have been made to target this pathway for the treatment of heavily vascularized malignant tumors, however, there has been little success in finding effective treatment options which strengthens the need for novel strategies [2,3].

Soft tissue sarcoma (STS) represents a rare malignant heterogeneous group of tumors that typically develops in the tissues that surround, connect, and support other body structures in adults [4]. Approximately 12,000 new cases of STS per year are seen in the United States, with ~50% of patients developing lung metastases, resulting in significant morbidity and mortality [5]. GBM is also a highly vascularized primary brain tumor in adults with extremely poor patient survival rate [2,3]. Therefore, there is an urgent need for novel therapies to improve the survival rate of both STS and GBM [6].

Herpes simplex virus-1-derived oncolytic viruses (oHSVs) are promising anticancer therapeutics, that can selectively replicate and lyse tumor cells and induce an immune response [7]. In addition to tumor cell killing, the development of an antitumor immune response is thought to be a key mechanism that underlies the efficacy of Imlygic, an oHSV-based therapeutic currently approved for melanoma [8]. Although oHSV are thought to infect and kill proliferating tumor endothelial cells in vitro and in vivo [9,10], these studies have either relied on in vitro assays or evidence of oHSV infection in tumor endothelium in vivo. To our knowledge, there has been no report on the role of tumor-associated endothelium infection on oncolysis. Apart from being an integral part of vasculature, endothelial cells also play an important role in the initiation, amplification, and resolution of the inflammatory response. We have previously shown that oHSV infection induces endothelial cell activation, resulting in a significant increase in blood vessel permeability and leukocyte adhesion [11]. It has also been reported that the residual tumors that occur after oHSV clearance become highly vascularized [12].

Vasculostatin (Vstat120) is an extracellular fragment of Brain Angiogenesis Inhibitor-1 (BAI1) that has potent antitumor and anti-angiogenic effects [13,14]. Rapid Antiangiogenesis Mediated By Oncolytic Virus (RAMBO) is a Vasculostatin-expressing oHSV [14]. In our previously studies, we showed that the RAMBO virus significantly enhance antitumor and anti-angiogenic efficacy in preclinical models of GBM, head and neck squamous cell carcinoma (SCC), and ovarian cancers through its ability to reduce angiogenesis [14,15,16]. Due to the prominent anti-angiogenic activity of RAMBO, we hypothesized that it would be also effective against highly vascularized malignant STSs. In this study, we found that sarcoma cells are highly susceptible to oHSV therapy and effectively express and secret Vstat120 after RAMBO virus infection. We used intravital in vivo imaging to examine the effect of oHSV on tumor vasculature and observed that tumor-associated endothelial cells hinder virus replication. However, RAMBO virus significantly reduced angiogenesis, and endothelial cell activation, both in vitro and in vivo, thereby enhancing antitumor efficacy and prolonging mice survival in subcutaneous sarcoma-bearing mice xenografts. This study reports on the significance of Vasculostatin-expressing oHSV therapy for the treatment of cancers.

## 2. Results

### 2.1. Co-Culture oHSV-Infected Tumor Cells with Endothelial Cells Reduces oHSV Replication

**Figure 1:**
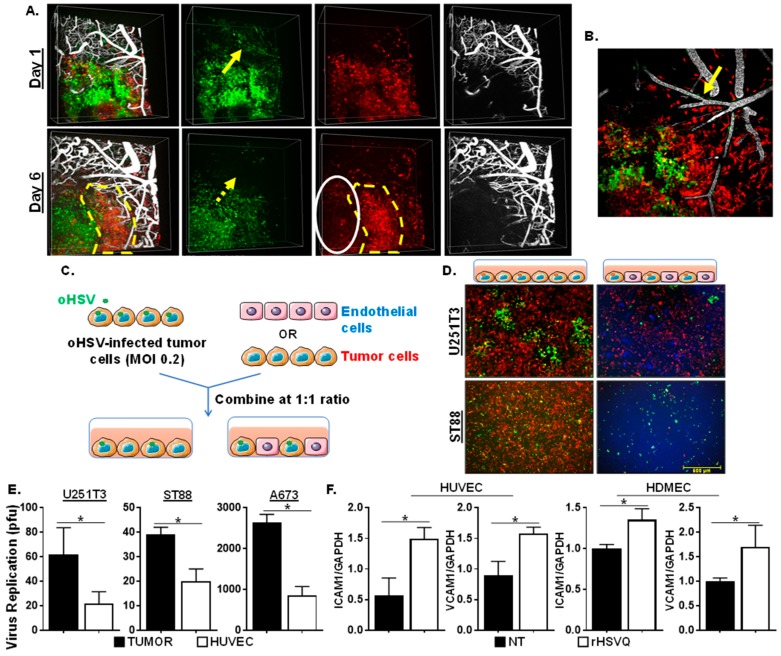
Endothelial cells reduce oncolytic herpes simplex virus-1 (oHSV) replication. (**A**) Intravital imaging revealed oHSV infection of tumor cells away from and close to vessels (yellow arrow) one day post-infection. On day 6 post viral infection, GFP signal in cells lining the vasculature had disappeared (yellow dot arrow) but, tumors not adjacent to vasculature (white oval) had sutained GFP-positive virus presence and reduced RFP-positive tumor cells over time. (**B**) Maximum Intensity Projection of inset shown in panel (**A**). Yellow arrow indicated oHSV-infected cells along perfused blood vessels. Green: oncolytic HSV-1 (GFP), Red: GBM12-RFP, White: Alexa Fluor 647-conjugated BSA. (**C**) Schematic diagram of experimental design for co-culture assay with tumor and endothelial cells. Sarcoma cells that stably express mCherry were infected with oHSV (rHSVQ: GFP) for an hour before being overlaid on either endothelial cells, which were stained with Cell Tracker Blue or mCherry expressing sarcoma cells at a 1:1 ratio. (**D**) Images were taken 24 h after infection to compare GFP expression in sarcoma cocultured with endothelial or sarcoma cells. (**E**) Viral titers were measured by standard plaque forming unit assay 24 h post-infection. (**F**) Human umbilical vein endothelial cells (HUVEC) and human dermal microvascular endothelial cells (HDMEC) were plated and treated with rHSVQ (MOI = 0.2) on 12-well plates. Quantitative real-time PCR (Q-PCR) analysis was done to examine for changes in gene expression of ICAM1 and VCAM1 between rHSVQ and Rapid Antiangiogenesis Mediated By Oncolytic Virus (RAMBO) infected endothelial cells 24 h post-infection. NT = No Treatment, * *p* < 0.05.

We have previously shown that oncolytic herpes simplex virus-1 (oHSV) treatment in mice bearing highly vascularized GBM tumors induces endothelial cell activation, thereby increasing blood vessel permeability and leukocyte adhesion [11]. To evaluate the spread of oHSV in tumors, we utilized intravital imaging of intracranial RFP-expressing primary GBM12 (GBM12-RFP) tumors in mice. These mice were infected with GFP-expressing control oHSV (rHSVQ), and vasculature was visualized by perfusion with 1% Alexa Fluor 647-conjugated BSA fluorescent dye given intravenously at the time of imaging. An efficient infection and spread of oHSV (visualized by GFP-positive infected cells) was obvious one day post-virus treatment. Areas of tumor not adjacent to vasculature (white oval) showed sustained virus presence and reduced RFP-positive tumor cells over time in vivo. Six days post-infection, GFP-positive virus-infected tumor cells could still be visualized in the RFP-positive tumor cells in vivo. However, tumor cells adjacent to tumor vasculature, appeared to have cleared the virus infection to allow for regrowth of GFP-negative and RFP-positive tumor cells (indicated by the yellow dotted line). Consistent with previous reports [17], we observed some infected cells lining perfused blood vessels on day one post-infection. Contrary to the suggestion that this reflects direct oncolytic destruction of tumor vasculature, we observed that by day six post-infection these cells became GFP negative, but remained viable and supported vessel perfusion, indicating the ability of infected cells lining vasculature to clear virus in vivo (Figure 1A,B).

To further evaluate the effects of endothelial cells on oHSV replication in tumor cells, we designed a tumor-endothelial cell co-culture system (Schematic diagram in Figure 1C). Stably mCherry-expressing human glioma (U251T3-mCherry) or soft tissue sarcoma (STS) (ST88-mCherry) cells were infected with GFP-expressing control oHSV (rHSVQ). Thirty minutes post-infection, unbound viruses were removed and overlaid on top of the equal number of either human umbilical vein endothelial cells (HUVEC) or tumor cells (U251T3-mCherry and ST88-mCherry) and cultured for 24 h (Figure 1C). Figure 1D shows a significant decrease in GFP positive virus-infected cells in the HUVEC cells overlaid with rHSVQ-infected tumor cells compared to the cells overlaid with tumor cells, indicating that endothelial cells display reduced viral replication in vitro. Consistent with fluorescent microscopy of GFP-positive-infected cells, quantification of virus replication also revealed a significant decrease in virus production in both GBM and sarcoma cells co-cultured with endothelial cells compared to cells co-cultured with tumor cells (U251T3 (2.86-fold, *p* < 0.05), ST88 (1.95-fold, *p* < 0.05), or A673 (3.08-fold, *p* < 0.05)) (Figure 1E). Increased expression of ICAM1 and VCAM1 on endothelial cells are well-known markers of endothelial cell activation [18,19]. To evaluate whether oHSV infection induces the expression of ICAM1 and VCAM1 on endothelial cells, we infected HUVEC and human dermal microvascular endothelial cells (HDMEC) with rHSVQ (MOI = 1) and measured changes in ICAM1 and VCAM1 gene expression using quantitative real-time PCR (Q-PCR) analysis (Figure 1F). There was a significant increase in gene expression of both ICAM1 and VCAM1 by rHSVQ infection, indicating that decreased virus replication in coculture with endothelial cells may be correlated with endothelial cell activation (Figure 1F). Collectively, these results showed that proliferating tumor endothelial cells can mount a potent antiviral effect that can limit virus spread in vitro and in vivo.

### 2.2. RAMBO Decreases Endothelial Cell Activation and Increases Viral Replication In Vitro

**Figure 2:**
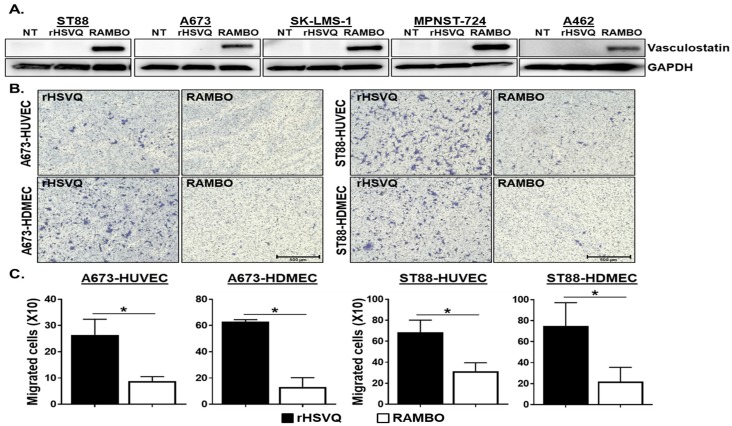
RAMBO expresses Vasculostatin in infected sarcoma cells and reduces endothelial cell migration. (**A**) RAMBO virus expresses Vasculostatin. Sarcoma cell lines (ST88, A673. SK-LMS-1, MPNST-724, and A462) were plated on 6 well plates and infected with 1 MOI of rHSVQ and RAMBO virus. The cells were harvested 24 h post-infection and analyzed by western blot for expression of Vasculostatin. Glyceraldehyde-3-phosphate dehydrogenase (GAPDH) was used as a loading control. Note the expression of Vstat120 in cells infected with RAMBO. NT = No treatment. (**B**) Sarcoma cell lines A673 and ST88 were treated with 1 MOI of rHSVQ and RAMBO for one hour and unbound virus was removed. CMs were collected 14 h post-infection and added to the bottom chamber of plate. Serum starved HUVEC and HDMEC cells were plated on the top chamber and were allowed to migrate for 6 h. Cells were then fixed and stained with Crystal Violet. Representative images of migrated endothelial cells (×100). (**C**) Migrated cell number was counted and compared between HSVQ and RAMBO infected cell conditions. Data are represented as mean ± SEM of number of cells/view field. * *p* < 0.05.

Vasculostatin (Vstat120) is a proteolytic fragment of brain angiogenic inhibitor 1 (BAI1) and has an anti-angiogenic and antitumorigenic activity [20]. To examine the impact of Vasculostatin expression from sarcoma cells infected with RAMBO on endothelial activation, we first tested the expression of Vasculostatin in sarcoma cells infected with RAMBO or control rHSVQ virus (Figure 2A). Western blot analysis on the lysates from ST88, A673, SK-LMS-1, MPNST-724, and A462 cells treated with control rHSVQ or RAMBO virus showed significantly increased expression of Vasculostatin in RAMBO-infected sarcoma cells (Figure 2A). Whole membrane scans of the Western blotting are shown in Figure S1. Next, we tested the sensitivity of sarcoma cells to oHSV-mediated killing efficacy by MTT assay in a panel of sarcoma tumor cells. Sarcoma cell viability was measured three days post-infection with control rHSVQ virus at the indicated MOI. Data were normalized to untreated cells at the same time point. Out of 5 sarcoma cells, A673 and ST88 cells were highly susceptible to oHSV (Figure S2). Thus, using A673 and ST88 cells, we tested the functionality of Vasculostatin produced by RAMBO-infected sarcoma cells (Figure 2B,C). Vasculostatin has been previously shown to inhibit endothelial cell migration, thus we evaluated the effect of conditioned medium (CM) from sarcoma cells infected with RAMBO or control rHSVQ virus on migration of endothelial cells. Treatment with CM collected from two different RAMBO-infected sarcoma cells significantly decreased the number of migrating HUVEC and HDMECs in a transwell assay (Figure 2B,C). Quantitative analysis showed that CM collected from A673 cells infected with RAMBO compared to rHSVQ treated significantly decreased the migration of HUVEC and HDMEC cells by 66.4% and 78.1%, respectively (Figure 2B, left). A similar effect was observed in the migration assay with CM from ST88 cells (HUVEC migration: 53.9%; HDMEC migration: 73.5%, *p <* 0.05) (Figure 2B, right).

Activated endothelial cells upregulate ICAM1 and VCAM1 to induce adhesion of leukocytes [18,19].

**Figure 3:**
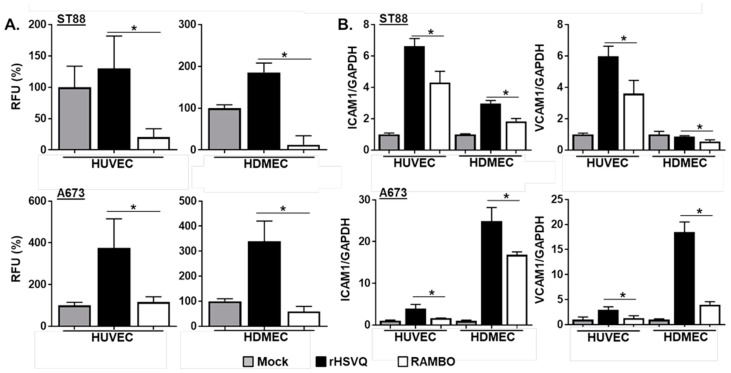
RAMBO decreases endothelial cell activation. (**A**) RAMBO decreases endothelial cell activation. HUVEC or HDMEC cells were plated on 96-well plates and treated with conditioned media (CM) collected from mock-, rHSVQ-, or RAMBO-infected sarcoma cells. Eight hours post CM treatment, cells were treated with LeukoTracker^TM^-labeled peripheral blood mononuclear cells (PBMCs) and allowed to adhere to endothelial cells. After 1 h incubation, unadherent PBMCs were washed off and adherent cells were lysed and quantified as instruction. Data is represented as relative fluorescence unit (RFU). (**B**) RAMBO decreases ICAM1 and VCAM1 gene expression. HUVEC and HDMEC cells were plated on 12-well plates. Six hours later, sarcoma cell lines were infected with 0.2 MOI of HSVQ and RAMBO for 30 min in a suspension condition. HUVEC and HDMC cells were overlaid with infected sarcoma cells and cultured for 24 h. Q-PCR analysis was conducted to examine for changes in gene expression of ICAM1 and VCAM1 between rHSVQ- and RAMBO-infected cells cocultured with endothelial cells. Data presented are fold changes in gene expression ± SD relative to GAPDH. * *p* < 0.05.

To examine the effect of RAMBO on the endothelial–leukocyte interactions, endothelial cells were treated with CM collected from mock-, rHSVQ-, or RAMBO-infected sarcoma cells and cultured for 8 h to activate endothelial cells. Then, cells were treated with fluorescence-labeled peripheral blood mononuclear cells (PBMCs) and allowed to adhere to endothelial cells. After 1 h incubation, unadherent PBMCs were washed off and total fluorescence attached to endothelial cells was measured (Figure 3A). A significant increase in fluorescence attached to endothelial cells was observed in rHSVQ-infected CM-treated cells. However, treatment with RAMBO-infected CM rescued the oHSV-induced increase in endothelial cell adhesion. As increased PBMC adhesion to endothelial cells is reflective of their activation [21], these results showed that RAMBO virus significantly decreases endothelial cell activation. Consistent with this, real-time PCR for ICAM1 and VCAM1 gene expression in endothelial cells overlaid with control rHSVQ- or RAMBO-infected sarcoma cells showed a significant decrease in ICAM1 and VCAM1 induction in the endothelial cells co-cultured with RAMBO virus-infected sarcoma cells compared to rHSVQ-infected cells (Figure 3B).

### 2.3. RAMBO Virus Increases Virus Replication and Endothelial Cell Killing

**Figure 4:**
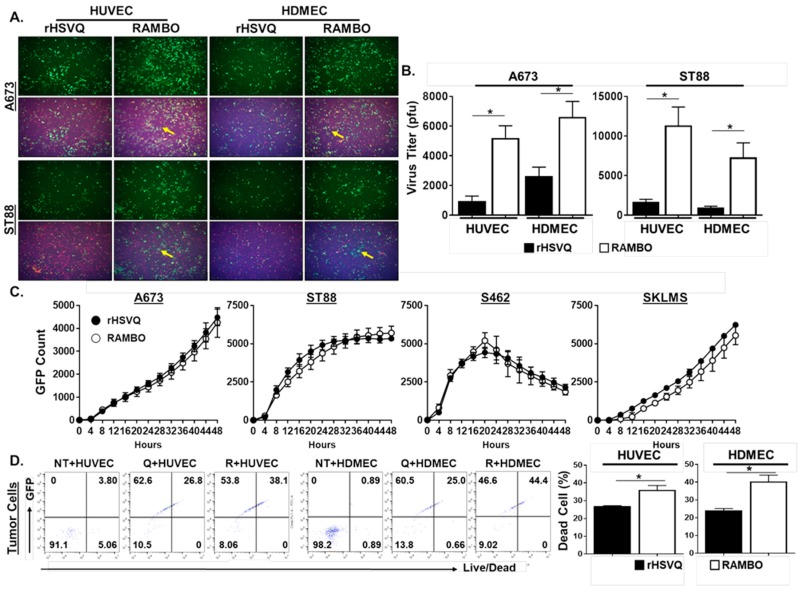
RAMBO increases virus replication in sarcoma and endothelial cell coculture condition. (**A**) Representative fluorescence microscopy images of GFP-positive virus-infected sarcoma cells cocultured with ECs. ECs were plates on 12 well plates. Six hours later, sarcoma cell lines A673 and ST88 were infected with 0.2 MOI oHSV (rHSVQ:GFP) and RAMBO for 30 min in a suspension condition. ECs were overlaid with infected mCherry-expressing sarcoma cells and cultured. Images were taken 24 h post-infection to compare GFP expression in RAMBO-infected cocultured cells vs HSVQ infected cells. ECs were stained with CellTracker Blue to distinguish between cell populations. (**B**) Quantification of virus replication. ECs were plated on 6 well plates. Six hours later, sarcoma cell lines A673 and ST88 were treated with 1 MOI of rHSVQ and RAMBO in a suspension condition. ECs were overlaid with infected sarcoma cells and cultured. Viral titers were measured by standard plaque forming unit assay 24 h post-infection and compared between rHSVQ and RAMBO infected cells. * *p* < 0.05. (**C**) Sarcoma cell lines were plated on 24 well plates and treated with rHSVQ and RAMBO virus for one hour. Unbound virus was removed and plate was imaged for 48 h using the Cytation 5 live plate reader/imager. Viral propagation was compared between rHSVQ and RAMBO infected cells via GFP expression. (**D**) Endothelial cells (ECs) were plates on 12-well plates. Six hours later, sarcoma cell line A673-mCherry was infected with 0.2 MOI rHSVQ and RAMBO for 30 min in a suspension condition. ECs were overlaid with infected A673-mCherry and cultured for 24 h. Cells were collected and stained with LIVE/DEAD fixable aqua cell stain. Flow cytometry was done to compare cell killing between rHSVQ- and RAMBO-infected cells and dead cell population was quantified and compared between groups. * *p* < 0.05.

To examine the effect of Vasculostatin on endothelial cell activation induced by oHSV infection, we overlaid rHSVQ- or RAMBO-infected sarcoma cells with the same number of endothelial cells. When oHSV-infected sarcoma cells were overlaid with endothelial cells, more GFP-positive virus-infected cells were obvious in RAMBO infected co-cultures compared to rHSVQ-infected cocultures (Figure 4A). This effect translated over to increased virus replication as higher viral titers were obtained when endothelial cells were overlaid with RAMBO-infected sarcoma cells relative to rHSVQ-infected cultures (Figure 4B). To further validate the RAMBO virus effect on endothelial cells, various sarcoma cells were infected with either control rHSVQ or RAMBO virus and viral spread was followed in cultures through quantification of virus-encoded GFP using a Cytation 5 Cell Imaging over time (Figure 4C). In the absence of endothelial cells, there was no significant difference in virus propagation between rHSVQ and RAMBO. Consistent with the Cytation result, there was no significant difference in virus titers between rHSVQ and RAMBO virus treated sarcoma cells in the absence of endothelial cells (Figure S3). We then examined the effects of RAMBO virus on tumor cell killing in an endothelial cell coculture condition. Using flow cytometry analysis, we observed that RAMBO virus-infected tumor cells in the co-cultures increased tumor cell killing compared to control rHSVQ-infected tumor cells in coculture (Figure 4D; relative to rHSVQ, 1.33-fold increase in co-culture with HUVEC and 1.68-fold increase in co-cultured with HDMEC, *p* < 0.05).

### 2.4. RAMBO Virus Reduces Angiogenesis and Enhances Antitumor Efficacy in Sarcoma Xenografts

**Figure 5:**
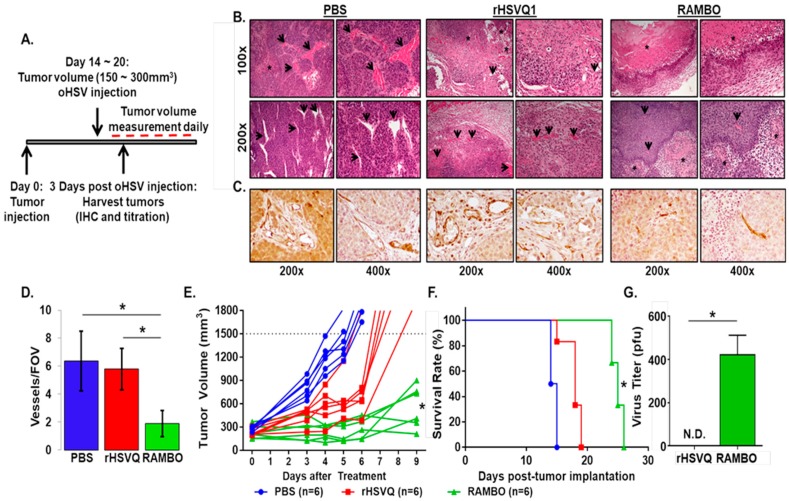
RAMBO virus reduces angiogenesis and enhances antitumor efficacy in sarcoma xenografts. (**A**) Schematic representation of animal study. All animal studies were conducted in the same way as shown in (**A**) except the time of tumor harvest. (**B**) Evaluation of antitumor and anti-angiogenic activity of RAMBO in vivo. Subcutaneous A673 sarcoma tumor-bearing mice were treated with PBS, rHSVQ or RAMBO and sacrificed three days after the virus treatment to harvest their tumor tissues for histological analysis. Hematoxylin and Eosin (H&E) staining was done on tumor tissues to compare areas of necrosis between treatment groups. Black arrows indicate endothelial cells and asterisks (*) indicate areas of necrotic tissue. (**C**) Representative images of tumor sections stained with CD31 antibody. Note significant reduction in CD31 positive tumor vasculature after RAMBO treatment compared to rHSVQ or PBS controls (*n* = 3/group). (**D**) Quantification of CD31 immunostained vessels per field of view in the tissue sections from treated sarcoma tumors was examined between treatment groups. Data shown are average vessel density per field of view of ×400 (FOV) ± SD (* *p* < 0.05). (**E**) Tested the antitumor efficacy of RAMBO in A673 sarcoma-bearing mice xenografts. Athymic nude mice with subcutaneous A673 sarcoma tumors were injected with rHSVQ1, RAMBO, or PBS by intratumoral injection when the A673 tumor volumes reached an average size of 150–300 mm^3^, and then tumor growth was measured daily. (**F**) Mice were sacrificed when tumor volume reached ≥1500 mm^3^ and mice survival was analyzed by Kaplan–Meier survival curve. (**G**) A673 tumors treated with HSVQ or RAMBO were harvested 3 days post virus treatment and dissociated and processed for titration. Viral titers were measured by standard plaque forming unit assay on Vero cells and compared between HSVQ and RAMBO infected tumors. N.D., not determined; * *p* < 0.05.

To evaluate the in vivo significance of RAMBO treatment, subcutaneously implanted A673 sarcoma tumors in athymic nude mice were treated with PBS, rHSVQ or RAMBO as shown in the schematic in Figure 5A. Mice were sacrificed three days post virus treatment, and tumor tissues were harvested for histological analysis (Figure 5A–C). Hematoxylin and Eosin (H&E) staining shows significantly increased areas of necrosis in the RAMBO-treated tumors (Figure 5B). Consistent with the increased necrosis, immunohistochemical staining (IHC) against an endothelial cell marker CD31 revealed a significant reduction of CD31-positive tumor vasculature in the RAMBO-treated tumors compared to rHSVQ- or PBS-treated tumors (Figure 5C). Furthermore, quantification of microvessel density (MVD) per field of view (FOV) in the tissue sections revealed 3.37- and 3.06-fold reduction in MVD in tumors treated with RAMBO compared to PBS or rHSVQ, respectively (*p* < 0.001) (Figure 5D).

Next, we tested the antitumor efficacy of RAMBO in A673 sarcoma-bearing mice xenografts. Athymic nude mice with subcutaneous A673 sarcoma tumors were injected with 5.5 × 10^6^ pfu of PBS, rHSVQ, or RAMBO by intratumoral injection when the A673 tumor volumes reached an average size of 150–300 mm^3^, and tumor growth was measured daily and monitored for changes in tumor size (Figure 5E). Control mice, which received PBS, showed rapid tumor growth with a tumor volume of 1408.18 ± 98.76 mm^3^ by day 6. In contrast, the mean tumor volumes in the mice treated with rHSVQ or RAMBO were 649.98 ± 104.98 and 241.20 ± 49.93, respectively. The average tumor volume differences revealed that a single intratumoral injection of RAMBO was enough to significantly decrease the tumor growth compared to PBS or rHSVQ (Figure 5E, *p* < 0.05). Mice were sacrificed when the tumor volume reached ≥1500 mm^3^, and mice survival was analyzed by Kaplan–Meier survival curve (Figure 5F). Overall mice survival rate was significantly prolonged by one injection of RAMBO with a median survival of 25 days compared to the 18.5 or 14 days in the rHSVQ or PBS group, respectively (*p* < 0.05; Figure 5F). More importantly, consistent with increased virus replication in the RAMBO virus-infected cells in co-culture with endothelial cells, there was a significant increase in virus titer in the RAMBO virus-treated tumor in in vivo (*p* < 0.05; Figure 5G). Taken together, there was a statistically significant therapeutic advantage of treating sarcoma-bearing mice with RAMBO.

## 3. Discussion

Tumor vascularization is a key characteristic that plays a role in sustaining tumor growth and metastasis. Therefore, it has become an attractive target in the development of cancer therapeutics [22]. Currently, there are a variety of anti-angiogenesis agents in clinical trials for heavily vascularized tumors including glioblastoma and soft tissue sarcomas (STSs) [23]. Nintedanib is an orally available agent being investigated in a phase I/II randomized trial in combination with immunotherapy drug Ipilimumab in Non Small Cell Lung Cancer (NSCLC) (NCT 03377023) as well as with cyclophosphamide in ovarian cancer patients (NCT 01610869). A tyrosine kinase inhibitor that targets VEGFR-2 (Apatinib) is also under investigation and has shown to prolong progression free survival (NCT 03491371) [24,25]. These, along with other antiangiogenesis drugs, are being combined with other agents to improve efficacy. With the recent FDA approval of the oncolytic herpes simplex virus-1 (oHSV), Talimogene Laherparepvec (T-Vec), for melanoma, developing methods to improve oHSV therapeutic efficacy is vital in order for it to become a therapeutic option for patients with highly vascularized tumors. However, the combination of angiogenesis inhibitors with oHSV therapy has not been tested for STS. In this study, using heavily vascularized STSs, we investigated a strategy to block angiogenesis and endothelial cell activation as treatment option for highly vascularized tumors by using oHSV therapy.

Various oncolytic viruses (OVs) including oHSV can infect and lyse proliferating tumor cells and endothelial cells, resulting in reduced perfusion and anti-angiogenic effects [17,26,27]. However, we and others have uncovered that OV treatment induces secretion of VEGF and IL-8 in the tumor microenvironment (TME), thereby increasing angiogenesis in residual tumors after virus clearance [28,29,30]. Combination treatment of OV with other angiogenesis inhibitors have shown overall enhancement in antitumor efficacy, but has not yet been tested in an STS model. We have shown that RAMBO virus, an oHSV expressing an anti-angiogenic gene *Vasculostatin*, reduces endothelial cell migration and angiogenesis, thereby aiding in the reduction of tumor growth. In addition, we have shown that RAMBO virus also reduces an oHSV therapy-induced innate inflammatory response in part through the suppression of TNFα secretion from macrophage/microglia [31,32]. This allows for an enhancement in virus replication and longer viral presence in in vivo than control viruses (rHSVQ). In this study, we evaluated the effect of RAMBO virus on endothelial cell activation and antitumor efficacy in heavily vascularized STSs.

In addition to the direct killing of tumor and tumor endothelial cells, OV therapy activates innate and adaptive antitumor immunity, thereby inflaming the cold tumor into hot TME. Although oHSV replication and propagation can be limited by the antiviral innate immune response, the initial local tumor cell killing by virus infection can reverse the immunosuppressive TME, thereby resulting in tumor associated antigen (TAA) release, cross-presentation, and antitumoral T cell recruitment [31,32]. Therefore, fine-tuning of oHSV-triggered early innate immune responses by effectively increasing the virus propagation and antitumor immune response is important to maximize the therapeutic efficacy of oHSV.

Inflammation is a basic cellular process in innate and adaptive immunity. Vascular endothelial cells play an important role in the initiation, amplification, and resolution of the inflammatory response. The leukocyte adhesion cascade involves the molecular interactions between leukocytes and endothelial cells and eventually allow for transendothelial migration by the leukocyte [33]. The up-regulation of adhesion molecules such as ICAM1 and VCAM1 by tumor vasculature plays a critical role in the recruitment of leukocytes to the tumor tissue and activation of endothelial cell [34,35]. oHSV therapy-induced ICAM1 induction on endothelial cells are also implicated in endothelial cell activation and inflammation [11]. Pro-angiogenic factors that are released into the TME play a role in manipulating endothelial cells to allow for leukocyte recruitment. The tumor vasculature is often poorly functional due to permeable and leaky vessels from endothelial junctional defects and pro-angiogenic signaling in the TME, thereby preventing efficient immune cell infiltration. These mechanisms have been noticed as limiting factors of current immunotherapies.

Our study shows that RAMBO viruses inhibit the activation of angiogenesis and anti-viral inflammation, resulting in better virus replication in tumor cells. Despite the benefit of viral replication through the inhibition of endothelial cell activation, it may also prevent infiltrating leukocytes, such as cytotoxic T cells, from reaching the tumor and reducing an antitumor immune responses. In future studies, it would be worthwhile to examine this caveat by implanting a tumor in immunocompetent mouse as xenograft models and determine the effects of RAMBO on immune cells recruitment in the tumor microenvironment.

Another STS treatment option to explore would be the use of immune checkpoint inhibitors (ICPIs) in combination with anti-angiogenic therapies. In the past decade, the development of immune checkpoint inhibitors (ICPIs) such as PD-1/PD-L1 and CTLA-4 inhibitors provided a breakthrough in the cancer therapies as evident by the increasing FDA approvals in recent years. Although some of the ICPIs have been remarkably successful for patients with a certain type of advanced cancers that have been resistant to standard therapy, ICPIs are only beneficial to the small fraction of cancer patients. Therefore, combination treatment of ICPIs with anti-angiogenic therapies are being heavily studied to improve the therapeutic efficacy of both treatments in multiple clinical trials. In the future, it would be interesting to explore by combining RAMBO virus with ICPIs such as PD1 or PD-L1 inhibitors for STSs.

## 4. Materials and Methods

### 4.1. Ethics Statement

All animal studies were approved by the Center for Laboratory Animal Medicine and Care (CLAMC) at The University of Texas Health Science Center at Houston (AWC-18-0059, 26 June 2018).

### 4.2. Cells and Viruses

Human soft tissue sarcoma cell lines (MPNST-724, A673, SK-LMS-1, ST88, and A462), U251T3 glioma, and Vero cells were maintained in Dulbecco’s Modified Eagle’s Medium (DMEM; Gibco BRL, Grand Island, NY, USA) supplemented with 10% fetal bovine serum (FBS). A673, SK-LMS-1, MPNST-724, ST88, and A462 cells were obtained from Dr. Raphael Pollock (The James Comprehensive Cancer Center, Ohio State University, Columbus, OH, USA) [36]. U251T3 was created by serially passaging U251MG cells in mice three times [20]. Patine-derived primary GBM cell (GBM12) were obtained from Mayo Clinic and cultured as tumor spheres in DMEM/F12 (Gibco, Waltham, MA) supplemented with B27 (Gibco), human EGF (20 ng/mL, R&D Systems, Minneapolis, MN), FGF (20 ng/mL, R&D Systems), sodium pyruvate (Gibco), 100 units/mL penicillin, and 0.1 mg/mL streptomycin (Gibco). Primary umbilical vein endothelial cells (HUVEC) and human dermal endothelial cells (HDMEC) were purchased from Sciencell and cultured in Endothelial Cell Medium (ECM; Sciencell, San Diego, CA, USA) as previously described [14]. Monkey kidney epithelial derived Vero cells were obtained from ATCC. STS and GBM cells were obtained from ATCC and authenticated by the University of Arizona Genetics Core via STR profiling in 2015. For oncolytic HSV, we used rHSVQ1 and rHSVQ-IE4/5-Vstat120 (RAMBO), which are both disrupted in the UL39 locus and deleted for both copies of the ICP34.5; RAMBO also contains the Vstat120 transgene under the HSV-1 IE4/5 promoter [37]. Viral stocks were generated and tittered on Vero African green monkey kidney cells (American Type Culture Collection, Manassas, VA, USA) via a standard plaque forming unit assay as previously described [20].

### 4.3. Co-Culture Assay for Viral Replication Assay

Primary HDMEC cells and HUVEC were plated in 6-well plates. Six hours later STS cells (A673 and ST88) were infected with virus at a multiplicity of infection [38] of 1 in the suspension condition. Thirty minutes after infection, unbound virus was removed and washed with PBS, and then cells were counted and overlaid onto HUVEC or HDMEC cells (1:1 ratio of HDMEC to STS cells). Twenty-four hours after co-culture, cells and media were collected, frozen, and thawed three times to release the viruses. The number of infectious particles present in the resulting supernatant was determined by performing a standard plaque formation assay on Vero cells as described [20]. All assays were performed in triplicate.

### 4.4. Flow Cytometry

Endothelial cells (ECs)—HUVEC or HDMECs—were plated on 6-well plates. Six hours later, stably mCherry-expressing A673 human sarcoma cells (A673-mCherry) were collected and infected with 0.1 MOI rHSVQ or RAMBO virus for 30 min in a suspension condition. Cells were washed and overlaid on top of an equivalent number of ECs and cultured for 24 h. Cells were then collected and stained with Live/Dead Fixable Aqua Dead Cell Stain Kit (Life Technologies, Eugene, OR, USA) and analyzed in CytoFlex (Beckman Coulter, Brea, CA, USA). Single stain controls for each fluorochrome were prepared using cells or compensation beads (Invitrogen, Waltham, MA, USA) for compensation. Tumor and endothelial cells were initially separated by gating for mCherry positive population (550–650 nm of emission), and then further gated for GFP expression and Live/Dead cell staining-positive polulation. GFP expression represents virus infected cells while Live/Dead staining represents dead cell population. Data were analyzed using FlowJo v10.7 (FlowJo LLC, Ashland, OR, USA).

### 4.5. Endothelial Cell Migration Assay

A673 and ST88 sarcoma cells were infected with the indicated virus at an MOI of 1. One hour post-infection, unbound virus was washed away, and serum-free media was added. Fourteen hours post virus infection, conditioned media (CM) was harvested, treated with 0.4% of human IgG to neutralize contaminated oHSV, and centrifuged for hour at 13,000 rpm to pellet any virus in the media. Endothelial cell migration assays were performed using a modified Boyden chamber (8-µm pore size, from Corning Costar (Cambridge, MA, USA) similar to previous reports [20]. Migration of serum-starved HUVEC and HDMEC towards CM was measured using transwell chambers. HUVEC and HDMEC were plated in the upper chamber, and cells were allowed to migrate for six hours, at which point membranes were fixed in 1% glutaraldehyde and stained with 0.5% crystal violet; unmigrated cells were removed from top chamber (using cotton swab). Images of the membranes were obtained at 20× magnification, and quantified by counting 3 fields of view/well (*n* = 3/group).

### 4.6. Endothelial Cell Adhesion Assay

CM was collected as described in the above endothelial cell migration assay. Adhesion of human donor peripheral blood mononuclear cells (PBMCs) to HUVECs or HDMECs was conducted using CytoSelect leukocyte–endothelium adhesion assay kit (Cell Biolabs, San Diego, CA, USA) as per the manufacturer’s instructions.

### 4.7. Quantitative Virus Spread by Cytation 5 Live Imaging

STS cells were infected with different MOIs of rHSVQ and RAMBO. One hour post virus infection, unbound virus was removed, and virus-encoded GFP expression was monitored every two hours for forty-eight hours using the Cytation 5 live plate reader/imager (BioTek Instruments). Captured images were analyzed by BioTek Gen5 version 2.06 software program (BioTek Instruments, lnc., Winooski, VT, USA). GraphPad Prism version 7.03 (GraphPad Software, La Jolla, CA, USA) was used for curve fitting, LD50 calculations, and statistical analysis [31].

### 4.8. Western Blot Assays

Cell lysates were fractionated by SDS-PAGE and transferred to polyvinylidene difluoride (PVDF) membranes. Blocked membranes were then incubated with antibodies against cleaved PARP (Cell Signaling Technology, Waltham, MA, USA); BAI1 and GAPDH (Abcam, Cambridge, MA, USA), HRP-conjugated secondary anti-mouse antibody (GE Healthcare, Piscataway, NJ, USA), and HRP-conjugated secondary goat anti-rabbit antibody (Dako, Hamburg, Germany). All antibodies were diluted 1:1000 and the immunoreactive bands were visualized using enhanced chemiluminescence (ECL) (GE Healthcare, Piscataway, NJ, USA).

### 4.9. Animal Surgery

All animal experiments were housed and performed in accordance with and approval of the Subcommittee on Research Animal Care of the Ohio State University and the Animal Welfare Committee at University of Texas Health Science Center (AWC-18-0059, 26 June 2018). Athymic NCr-nu/nu mice, (specify male or female), outbred were provided by the OSUCCC Target Validation Shared Resource (TVSR); the original breeders, Strain number 553 and 554 were purchased either from the NCI Frederick Facility or Charles River. Four- to five-week-old female athymic nu/nu mice (NIH-NCI) were injected subcutaneously with 2 × 10^6^ of A673-SCC-74A with matrigels (A673:matrigel = 50 μL: 50 μL) in 100 μL volume into the rear flank. When tumors reached average size of 150–200 mm^3^, mice were randomized and 5.5 × 10^6^ pfu of oHSV (rHSVQ1 and RAMBO) or PBS was administered by direct intratumoral injection. The tumor volume was calculated using the following formula: Volume = 0.5 × L × W^2^, where *W* and *L* are the two maximum dimensions. Measurements of tumor volumes were taken as indicated, and mice were killed when tumor volumes exceeded 1500 mm^3^ or >20% of body mass was lost.

### 4.10. Intravital Imaging

Six- to eight-week old NSG mice were implanted with GBM12-RFP cells (2 × 10^5^ cells) stereotaxically into the right hemisphere (2 mm lateral and 2.5 mm posterior from bregma, 1 mm depth). 2 weeks later, mice were stereotaxically infected with rHSVQ (2 × 10^5^ pfu) and cranial window surgery was performed. Craniectomy was done over the tumor-implanted area, and the brain surface was covered with cover glass (Bioscience Tools, San Diego, CA, USA) which was glued to the skull with dental resin. For intravital imaging, mice were anesthetized and positioned on the stage of a confocal microscope (NIKON A1R-MP, NIKON, Tokyo, Japan). Then, 100 uL of 1% Alexa Fluor 647 conjugated BSA (Thermo Fisher Scientific, Waltham, MA, USA) was administrated through the tail vein. Images were acquired on day 1 and day 6 post viral infection.

### 4.11. Histological Analysis

Tumor tissues were fixed in 4% paraformaldehyde, embedded in paraffin and cut into 5-µm sections. Representative sections were stained with Hematoxylin and Eosin (H&E), and then examined by light microscopy. For immunohistochemistry of CD31, sections were stained using antimouse CD31 antibody at a 1:250 dilution (BD PharMingen, San Jose, CA, USA). Appropriate negative and positive controls were done. The total number of stained vessels was determined in five random high-power fields (×400 magnification), and the mean then was reported in a blinded fashion for each tumor.

### 4.12. Statistics

GraphPad software was used for all statistical analysis. Data are presented as mean ± SD. Data were analyzed using unpaired, two-tailed *t*-tests when comparing two variables. ANOVA with Tukey’s post-test was used to compare data in experiments where more than two variables were compared simultaneously. Log-rank test was used to compare survival curves for survival data. A *p* value 0.05 or less is considered significant.

## 5. Conclusions

Herpes simplex virus-1-derived oncolytic viruses (oHSVs) promise the development of novel biological therapeutic strategies for many potential applications, as the oHSVs selectively replicate in tumor cells to lyse and induce a concurrent immune response. In this study, we examined the effect of oHSV treatment on tumor vasculature by using intravital in vivo live imaging and observed that tumor-associated endothelial cells hinder virus replications, which can be rescued by oHSV expressing an anti-angiogenic gene Vasculostatin, RAMBO (Rapid Anti-angiogenesis Mediated By Oncolytic virus). In addition, our results successfully demonstrated that treatment with RAMBO virus significantly decreased endothelial cell density in the TME, enhanced antitumor efficacy, and prolonged survival in mice inoculated with heavily vascularized A673 soft tissue sarcoma (STS) xenografts.

## Supplementary Materials

The following are available online at https://www.mdpi.com/2072-6694/12/4/1040/s1, Figure S1: RAMBO expresses Vasculostatin in infected sarcoma cells, Figure S2: Sarcoma cells are sensitive to killing by oHSV: A panel of sarcoma cells were plated on 96 well plates and treated with 5 different MOIs of rHSVQ and cultured for 72 h. Cell viability was measured by MTT assay to compared cell killing between uninfected and rHSVQ-infected cells, Figure S3: No significant difference in virus titers between rHSVQ and RAMBO virus treated human sarcoma cells in the absence of endothelial cells. A673 and ST88 were treated with 0.1 MOI of rHSVQ and RAMBO. One hour post infection, unbound virus were removed and cultured. Viral titers were measured by standard plaque forming unit assay 24 h post infection and compared between rHSVQ and RAMBO infected cells.

## References

[1] Mir O., Penel N. (2017). Targeting angiogenesis in advanced soft tissue sarcoma: Tivozanib-hype or me-too?. Ann. Oncol..

[2] Sborov D., Chen J.L. (2015). Targeted therapy in sarcomas other than GIST tumors. J. Surg. Oncol..

[3] Ricci-Vitiani L., Pallini R., Biffoni M., Todaro M., Invernici G., Cenci T., Maira G., Parati E.A., Stassi G., Larocca L.M. (2010). Tumour vascularization via endothelial differentiation of glioblastoma stem-like cells. Nature.

[4] Katz D., Palmerini E., Pollack S.M. (2018). More Than 50 Subtypes of Soft Tissue Sarcoma: Paving the Path for Histology-Driven Treatments. Am. Soc. Clin. Oncol. Educ. Book.

[5] Chudgar N.P., Brennan M.F., Munhoz R.R., Bucciarelli P.R., Tan K.S., D’Angelo S.P., Bains M.S., Bott M., Huang J., Park B.J. (2017). Pulmonary metastasectomy with therapeutic intent for soft-tissue sarcoma. J. Thorac. Cardiovasc. Surg..

[6] Siegel R.L., Miller K.D., Jemal A. (2015). Cancer statistics, 2015. CA Cancer J. Clin..

[7] Saha D., Wakimoto H., Rabkin S.D. (2016). Oncolytic herpes simplex virus interactions with the host immune system. Curr. Opin. Virol..

[8] Rothermel L.D., Zager J.S. (2018). Engineered oncolytic viruses to treat melanoma: Where are we now and what comes next?. Expert Opin. Biol. Ther..

[9] Cinatl J., Michaelis M., Driever P.H., Cinatl J., Hrabeta J., Suhan T., Doerr H.W., Vogel J.U. (2004). Multimutated herpes simplex virus g207 is a potent inhibitor of angiogenesis. Neoplasia.

[10] Mahller Y.Y., Vaikunth S.S., Currier M.A., Miller S.J., Ripberger M.C., Hsu Y.H., Mehrian-Shai R., Collins M.H., Crombleholme T.M., Ratner N. (2007). Oncolytic HSV and erlotinib inhibit tumor growth and angiogenesis in a novel malignant peripheral nerve sheath tumor xenograft model. Mol. Ther..

[11] Hong B., Muili K., Bolyard C., Russell L., Lee T.J., Banasavadi-Siddegowda Y., Yoo J.Y., Yan Y., Ballester L.Y., Bockhorst K.H. (2019). Suppression of HMGB1 Released in the Glioblastoma Tumor Microenvironment Reduces Tumoral Edema. Mol. Ther Oncolytics.

[12] Kurozumi K., Hardcastle J., Thakur R., Yang M., Christoforidis G., Fulci G., Hochberg F.H., Weissleder R., Carson W., Chiocca E.A. (2007). Effect of tumor microenvironment modulation on the efficacy of oncolytic virus therapy. J. Natl. Cancer Inst..

[13] Kaur B., Cork S.M., Sandberg E.M., Devi N.S., Zhang Z., Klenotic P.A., Febbraio M., Shim H., Mao H., Tucker-Burden C. (2009). Vasculostatin inhibits intracranial glioma growth and negatively regulates in vivo angiogenesis through a CD36-dependent mechanism. Cancer Res..

[14] Hardcastle J., Kurozumi K., Dmitrieva N., Sayers M.P., Ahmad S., Waterman P., Weissleder R., Chiocca E.A., Kaur B. (2010). Enhanced antitumor efficacy of vasculostatin (Vstat120) expressing oncolytic HSV-1. Mol. Ther..

[15] Yoo J.Y., Yu J.G., Kaka A., Pan Q., Kumar P., Kumar B., Zhang J., Mazar A., Teknos T.N., Kaur B. (2015). ATN-224 enhances antitumor efficacy of oncolytic herpes virus against both local and metastatic head and neck squamous cell carcinoma. Mol. Ther Oncolytics.

[16] Tomita Y., Kurozumi K., Yoo J.Y., Fuji K., Ichikawa T., Matsumoto Y., Uneda A., Hattori Y., Shimizu T., Otani Y. (2019). Oncolytic herpes virus armed with vasculostatin in combination with bevacizumab abrogate glioma invasion via the CCN1 and AKT signaling pathways. Mol. Cancer Ther.

[17] Toro Bejarano M., Merchan J.R. (2015). Targeting tumor vasculature through oncolytic virotherapy: Recent advances. Oncolytic Virother.

[18] Sans M., Panes J., Ardite E., Elizalde J.I., Arce Y., Elena M., Palacin A., Fernandez-Checa J.C., Anderson D.C., Lobb R. (1999). VCAM-1 and ICAM-1 mediate leukocyte-endothelial cell adhesion in rat experimental colitis. Gastroenterology.

[19] Szmitko P.E., Wang C.H., Weisel R.D., de Almeida J.R., Anderson T.J., Verma S. (2003). New markers of inflammation and endothelial cell activation: Part I. Circulation.

[20] Yoo J.Y., Haseley A., Bratasz A., Chiocca E.A., Zhang J., Powell K., Kaur B. (2012). Antitumor efficacy of 34.5ENVE: A transcriptionally retargeted and "Vstat120"-expressing oncolytic virus. Mol. Ther..

[21] Harjunpaa H., Llort Asens M., Guenther C., Fagerholm S.C. (2019). Cell Adhesion Molecules and Their Roles and Regulation in the Immune and Tumor Microenvironment. Front. Immunol..

[22] Farnsworth R.H., Lackmann M., Achen M.G., Stacker S.A. (2014). Vascular remodeling in cancer. Oncogene.

[23] Rocchi L., Caraffi S., Perris R., Mangieri D. (2014). The angiogenic asset of soft tissue sarcomas: A new tool to discover new therapeutic targets. Biosci Rep..

[24] Zhu B., Li J., Xie Q., Diao L., Gai L., Yang W. (2018). Efficacy and safety of apatinib monotherapy in advanced bone and soft tissue sarcoma: An observational study. Cancer Biol. Ther..

[25] Riezu-Boj J.I., Moriyon I., Blasco J.M., Marin C.M., Diaz R. (1986). Comparison of lipopolysaccharide and outer membrane protein-lipopolysaccharide extracts in an enzyme-linked immunosorbent assay for the diagnosis of Brucella ovis infection. J. Clin. Microbiol..

[26] Ungerechts G., Bossow S., Leuchs B., Holm P.S., Rommelaere J., Coffey M., Coffin R., Bell J., Nettelbeck D.M. (2016). Moving oncolytic viruses into the clinic: Clinical-grade production, purification, and characterization of diverse oncolytic viruses. Mol. Ther. Methods Clin. Dev..

[27] Fountzilas C., Patel S., Mahalingam D. (2017). Review: Oncolytic virotherapy, updates and future directions. Oncotarget.

[28] Kurozumi K., Hardcastle J., Thakur R., Shroll J., Nowicki M., Otsuki A., Chiocca E.A., Kaur B. (2008). Oncolytic HSV-1 infection of tumors induces angiogenesis and upregulates CYR61. Mol. Ther..

[29] Aghi M., Rabkin S.D., Martuza R.L. (2007). Angiogenic response caused by oncolytic herpes simplex virus-induced reduced thrombospondin expression can be prevented by specific viral mutations or by administering a thrombospondin-derived peptide. Cancer Res..

[30] Wojton J., Kaur B. (2010). Impact of tumor microenvironment on oncolytic viral therapy. Cytokine Growth Factor Rev..

[31] Yoo J.Y., Swanner J., Otani Y., Nair M., Park F., Banasavadi-Siddegowda Y., Liu J., Jaime-Ramirez A.C., Hong B., Geng F. (2019). oHSV therapy increases trametinib access to brain tumors and sensitizes them in vivo. Neuro Oncol..

[32] Bolyard C., Meisen W.H., Banasavadi-Siddegowda Y., Hardcastle J., Yoo J.Y., Wohleb E.S., Wojton J., Yu J.G., Dubin S., Khosla M. (2017). BAI1 Orchestrates Macrophage Inflammatory Response to HSV Infection-Implications for Oncolytic Viral Therapy. Clin. Cancer Res..

[33] Georganaki M., van Hooren L., Dimberg A. (2018). Vascular Targeting to Increase the Efficiency of Immune Checkpoint Blockade in Cancer. Front. Immunol.

[34] Fiuza C., Bustin M., Talwar S., Tropea M., Gerstenberger E., Shelhamer J.H., Suffredini A.F. (2003). Inflammation-promoting activity of HMGB1 on human microvascular endothelial cells. Blood.

[35] Lorenzon P., Vecile E., Nardon E., Ferrero E., Harlan J.M., Tedesco F., Dobrina A. (1998). Endothelial cell E- and P-selectin and vascular cell adhesion molecule-1 function as signaling receptors. J. Cell Biol..

[36] Lopez G., Bill K.L., Bid H.K., Braggio D., Constantino D., Prudner B., Zewdu A., Batte K., Lev D., Pollock R.E. (2015). HDAC8, A Potential Therapeutic Target for the Treatment of Malignant Peripheral Nerve Sheath Tumors (MPNST). PLoS ONE.

[37] Terada K., Wakimoto H., Tyminski E., Chiocca E.A., Saeki Y. (2006). Development of a rapid method to generate multiple oncolytic HSV vectors and their in vivo evaluation using syngeneic mouse tumor models. Gene Ther..

[38] De Wilde V., Van Rompaey N., Hill M., Lebrun J.F., Lemaitre P., Lhomme F., Kubjak C., Vokaer B., Oldenhove G., Charbonnier L.M. (2009). Endotoxin-induced myeloid-derived suppressor cells inhibit alloimmune responses via heme oxygenase-1. Am. J. Transplant..

