# Supplementary material for: Enhancing Antitumor Efficacy of Heavily Vascularized Tumors by RAMBO Virus through Decreased Tumor Endothelial Cell Activation
Source: Cancers (Basel). 2020 Apr 23;12(4):1040. doi: 10.3390/cancers12041040 (PMC7225935; doi:10.3390/cancers12041040)
Supplement: Supplementary file 1 [file cancers-12-01040-s001.pdf]

Supplementary Materials: Enhancing Antitumor Efficacy of Heavily Vascularized Tumors by RAMBO Virus through Decreased Tumor Endothelial Cell Activation

Mitra Nair, Maninder Khosla, Yoshihiro Otani, Margaret Yeh, Flora Park, Toshihiko Shimizu, Jin Muk Kang, Chelsea Bolyard, Jun-Ge Yu, Yeshavanth Kumar Banasavadi-Siddegowda, Gonzalo Lopez, Balveen Kaur, Raphael E. Pollock, Tae Jin Lee, Matthew Old and Ji Young Yoo

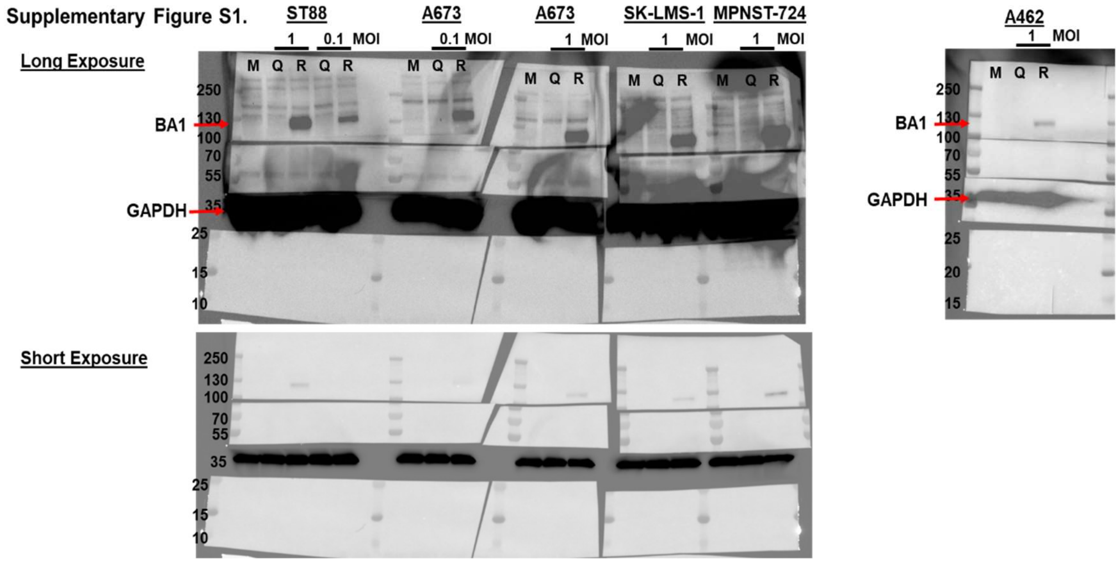

Figure S1. RAMBO expresses Vasculostatin in infected sarcoma cells. (**A**) RAMBO virus expresses Vasculostatin. Sarcoma cell lines (ST88, A673. SK-LMS-1, MPNST-724, and A462) were plated on 6-well plates and infected with 1 MOI of rHSVQ and RAMBO virus. The cells were harvested 24 h post-infection and analyzed by western blot for expression of Vasculostatin. Glyceraldehyde-3-phosphate dehydrogenase (GAPDH) was used as a loading control. Note the expression of Vasculostatin in cells infected with RAMBO. M: Mock; Q: rHSVQ; R: RAMBO.

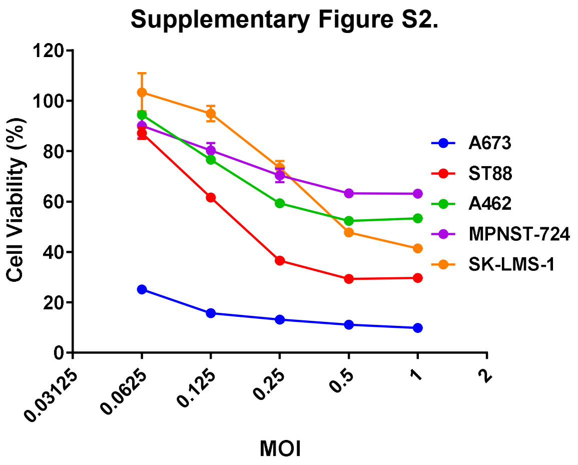

Figure S2. Sarcoma cells are sensitive to killing by oHSV: A panel of sarcoma cells were plated on 96-well plates and treated with 5 different MOIs of rHSVQ and cultured for 72 h. Cell viability was measured by MTT assay to compared cell killing between uninfected and rHSVQ-infected cells.

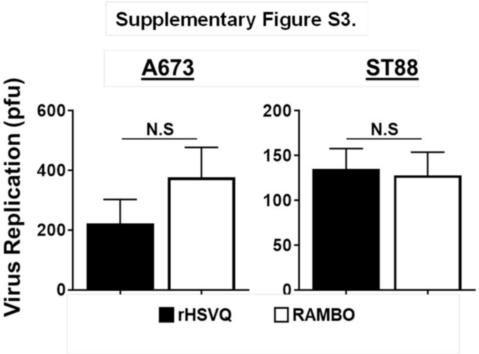

Figure S3. No significant difference in virus titers between rHSVQ and RAMBO virus treated human sarcoma cells in the absence of endothelial cells. A673 and ST88 were treated with 0.1 MOI of rHSVQ and RAMBO. One hour post-infection, unbound virus were removed and cultured. Viral titers were measured by standard plaque forming unit assay 24 h post-infection and compared between rHSVQ and RAMBO infected cells.

© 2020 by the authors. Licensee MDPI, Basel, Switzerland. This article is an open access article distributed under the terms and conditions of the Creative Commons Attribution (CC BY) license (http://creativecommons.org/licenses/by/4.0/).
